# Endothelial Function in Patients With Von Willebrand Disease

**DOI:** 10.1177/1076029620984546

**Published:** 2021-01-15

**Authors:** Stephanie Noone, Ralf Schubert, Stephan Fichtlscherer, Thomas Hilberg, Sonja Alesci, Wolfgang Miesbach

**Affiliations:** 1 Haemostaseology, Department of Internal Medicine II, Institute of Transfusion Medicine, University Hospital, Goethe University Frankfurt am Main, Frankfurt, Germany; 2 Department for Children and Adolescents Medicine, University Hospital Frankfurt, Frankfurt am Main, Germany; 3 Division of Cardiology, Department of Internal Medicine III, University Hospital Frankfurt, Frankfurt am Main, Germany; 4 Department of Sports Medicine, University of Wuppertal, Wuppertal, Germany; 5 IMD Blood Coagulation Centre, Bad Homburg, Germany

**Keywords:** von Willebrand disease, endothelial function, coronary artery disease, comorbidity

## Abstract

In patients with von Willebrand disease (vWD) the interest in age-related comorbidities has grown, because the life expectancy of these patients has increased. The research question of this study was whether patients with vWD show a different endothelial function compared to the general population. A total of 37 patients with type 1 (n = 23), type 2 (n = 10) and type 3 (n = 4) vWD, 14 controls and 38 patients with coronary artery disease (CAD) were included in this study. Five markers of endothelial dysfunction (MOED) were determined. Moreover, the endothelial function was examined using the Itamar Endo-PAT. The reactive hyperemia index (RHI) was calculated from the results. The markers soluble intercellular adhesion molecule-1 (p = 0.171), P-Selectin (p = 0.512), interleukin-6 (p = 0.734) and monocyte chemoattractant protein-1 (p = 0.761) showed higher levels in patients with vWD, but were not significantly different compared to the control group. RHI was impaired in CAD-patients (1.855), whereas vWD patients had mean results of 1.870 and controls 2.112 (p = 0.367). In this study, the endothelial function measurements of patients with von Willebrand disease were not significantly different compared to healthy controls.

## Introduction

The most common congenital bleeding disorder is von Willebrand disease (vWD). The mode of inheritance is autosomal-dominant (type 1 and 2) or autosomal-recessive (type 3). Patients suffer from a quantitative deficiency (type 1 and 3) or qualitative deficiency (type 2) of von Willebrand factor (VWF) which mediates the platelet adhesion and aggregation at the subendothelium. Moreover, the VWF acts as a support protein of factor VIII and occurs together with it in a complex. Therefore, patients with vWD often simultaneously have a factor VIII activity deficiency.^1^

Clinically, the vWD manifests mainly in the form of mucosal bleeding, especially nosebleeds, bleeding gums and in women menorrhagia, but also hemarthrosis and hematomas are possible. Severity is mainly mild in type 1 and increases in type 2 and 3. Bleedings in patients with type 1 are predominantly post-traumatic e.g. after dental surgery, whereas bleeding in type 3 patients is sometimes similar to hemophilia patients.^1^

Nowadays, patients with congenital bleeding disorders have an increased life expectancy, due to improved treatment options.^2^ Therefore, emphasis should be placed on the treatment of age-related comorbidities.

Of particular interest in vWD patients are cardiovascular diseases and, above all, coronary artery disease and related myocardial infarction complications. On the one hand, because cardiovascular diseases are among the leading causes of death worldwide.^3^ On the other hand, because it is a matter of debate whether the deficiency or abnormality of VWF and the factor VIII activity deficiency in patients with vWD protect against atherosclerosis and thrombus formation.

Coronary artery disease (CAD) is the manifestation of atherosclerosis in the coronary vessels beginning with endothelial dysfunction. The healthy endothelium has vasoprotective properties whereas endothelial dysfunction has the resulting effects such as increased leukocyte adhesion and promotion of inflammatory processes. It is described that atherosclerosis derives from a chronic inflammatory process in the vessels. Myocardial infarction can occur as a result of two mechanisms which are formation of a clinically relevant stenosis by an atherosclerotic plaque and acute thrombus formation after a plaque rupture.^4^

Damage to the endothelium due to risk factors such as hypertension and diabetes mellitus, leads to platelet adhesion and aggregation and activation of the coagulation cascade. The VWF is involved in both processes because of its role in mediating the adhesion of platelets to the subendothelium and in providing the stability of factor VIII, which is important in the coagulation cascade.^5^ A study from Alesci et al. documented that also patients with vWD suffer from arterial hypertension.^6^

Hence, the release of VWF is increased by endothelial damage. Therefore, VWF is also considered a marker of endothelial dysfunction. Several studies have confirmed that high levels of VWF are associated with coronary heart disease and ischemic stroke. Sonneveld et al. published a study in 2014 in which 581 patients with acute coronary syndrome or stable angina pectoris were included. High levels of VWF were shown to be prognostically unfavorable in a 1-year follow-up.^7^

This could also be due to the fact that the VWF plays a role not only in endothelial damage at the beginning of the development of atherosclerosis but also in manifest atherosclerosis. After the rupture of an atherosclerotic plaque, the VWF is involved in the thrombus formation.^8^

Furthermore, if patients with vWD have a factor VIII activity deficiency less thrombin is produced, which catalyzes the conversion from fibrinogen to fibrin for thrombus formation.^9^

New treatment options lead to rise in life expectancy of vWF patients. The existing literature on age-related comorbidities of patients with vWD is rare. That makes the topic currently important. Furthermore, several studies showed an association between high levels of vWF and endothelial damage/cardiovascular events. But only few studies have examined the effect of low vWF levels in human. The strength of this study will be, that endothelial function will be examined in in patients associated with low, normal and high levels of vWF.

Consequently, it can be hypothesized that decreased levels of VWF, as found in vWD, have a protective effect. Due to decreased platelet aggregation, the process of atherosclerosis in patients with vWD may be slower, and thus would have some protection against coronary heart disease, or it would occur clinically later.

An animal study with mice without VWF showed, that these mice develop less atherosclerosis.^8^ A review from van Galen et al. gives a detailed overview over the results from different studies with animal models with VWF deficiency or blockage.^10^ These studies suggest that VWF deficiency may have a protective effect on atherosclerosis at arterial branch point predilection sites and on arterial thrombosis.^10^

With respect to the increased life expectancy of patients with vWD and small amount of knowledge about the degree of atherosclerosis in patients with vWD, the aim of this study was to determine whether these patients have a different endothelial function than the general population due to their bleeding disorder. Therefore, laboratory measurements and non-invasive Endo-PAT were performed to determine endothelial function.

## Materials and Methods

Patients with vWD, healthy controls and patients with CAD were included in this pilot study. Inclusion criteria was that patients were older or equal 40 years of age. The 3 groups were matched for age. All test persons signed an informed consent and were examined at the University Hospital Frankfurt. Every vWD patient regularly visits the hemophilia treatment center of the hospital. The data of the CAD patients was kindly provided by the Division of Cardiology. This study has received a positive ethics committee vote and was performed after the GCP (Good Clinical Practice).

The data were initially collected in a study with even more patients. The same studies were also carried out in hemophilia patients. Therefore the following methods have already been reported in Böhmert et al. (2018).^11^

Demographic data, cardiovascular risk factors related to body mass index, arterial hypertension, diabetes mellitus type 2 and smoking were gathered and reviewed. Also, vWD specific information about vWD type and results from VWF antigen, VWF activity and FVIII activity measurements were collected. Moreover, blood pressure measurement, blood draw for measuring biochemical markers of endothelial dysfunction (MOED) and vWD specific parameters and the endothelial function measurement were performed.

The biochemical markers of endothelial dysfunction were soluble intercellular adhesion molecule-1 (sICAM-1), platelet selectin (P-selectin), interleukin-6 (Il-6), tumor necrosis factor alpha (TNF-α) and monocyte chemoattractant protein-1 (MCP-1).^4,12^ High levels of these markers are an indication for endothelial dysfunction. Their levels were measured in the patients’ blood serum with the Cytometric Bead Array (CBA) method. For the assays the BD (Becton Dickinson) FACSArray™ (Fluorescence-activated cell sorting) bioanalyzer and FCAP Array v3.0 Software (BD Biosciences, San Jose, CA, USA) were used. Details on the technique of this method have been reported in Eickmeier et al. (2010).^13^

Endothelial function was measured using the Itamar Endo-PAT (Endo-PAT 2000, Itamar Medical Inc., Caesarea, Israel). Most commonly in other studies and clinical practice the endothelial function is examined using the ultrasonography method called flow-mediated dilatation of the brachial artery. However, this method however is user dependent. The Endo-PAT method is userindependent and provides automatically calculated results for assessing endothelial function. This reduces variability within and across cohorts. Precisely, the Endo-PAT measures the endothelium-dependent reactive hyperemia index (RHI) of the digital arteries, which is a marker of endothelial function.^14^ The higher the RHI, the greater are the arterial pulsatile volume changes after releasing the occlusion of the blood supply to the finger, the better is the endothelial function and the lower is the risk for cardiovascular disease. The RHI results can be assigned to three different categories: a RHI ≥2.1 indicates a low, between 1.68 and 2 a moderate and ≤1.67 a high risk of cardiovascular disease.^15^

Statistical analysis was done using GraphPad Prism (Version 5, GraphPad Software, Incorporated, La Jolla, California, United States) and BiAS for Windows (Version 11.02—03/2016, epsilon-publisher, Dr. Ackermann, Goethe-University Frankfurt).

The Kolmogorov-Smirnov-test was used to test whether a data set was normally distributed. To compare 2 groups the t-test was used. If the data was not normally distributed the Mann-Whitney-test was applied. To compare 3 groups, the analysis of variance (ANOVA) test was used. If the data was not normally distributed, the Kruskal-Wallis-test together with Multiple Conover-Iman comparisons was applied. To show the impact of a low case number the power of a test was measured. To compare datasets with nominally scaled data sets the Chi^2^-test was used. To correlate the datasets Spearman´s correlation was applied.

## Results

This study included 37 patients with vWD and 14 healthy controls (without bleeding disorder). With 62% most of the vWD patients had vWD type 1 (Table 1). Both frequency and mean VWF activity decreased from type 1 to type 3. Furthermore, 38 patients with CAD were included. Blood samples of patients with CAD were not available, because none were taken by the Division of Cardiology. Therefore, the MOEDs could not be examined in CAD patients.

**Table 1.** vWD Patients: Characteristics of Different Types.

	**Number of patients**	**VWF antigen [%]**	**VWF activity** [%]	**FVIII activity** [%]
**vWD**	37	38.8 ± 20.9	26.4 ± 16.5	51.2 ± 27.2
**Type 1**	23 (62%)	48.7 ± 15.2	36.2 ± 11.8	66.0 ± 16.8
**Type 2**	10 (27%)	29.2 ± 18.1	12.1 ± 9.1	35.3 ± 22.4
**Type 3**	4 (11%)	5.5 ± 4.8	6.0 ± 6.2	5.9 ± 5.4

Indicated is the mean ± standard deviation of VWF antigen as well as VWF and FVIII activity; vWD von Willebrand disease, VWF von Willebrand factor.

Table 2 presents the study population’s demographic data and the prevalence of cardiovascular risk factors as well as infectious diseases and coronary artery disease. The mean age of vWD patients was 52.0 years, for controls 49.1 years and for CAD patients 51.2 years. The 3 groups had no equal gender distribution, because only data from 5 female CAD patients were available. The cardiovascular risk factors type 2 diabetes mellitus (vWD patients 8%, controls 0%); p = 0.666) and smoking (vWD patients 19%, controls 29%; p = 1.000) were not significantly different between vWD patients and controls. But blood pressure was significantly higher in vWD patients compared to controls and CAD patients (systolic blood pressure: mean ± standard deviation (sd): vWD patients 134.4 ± 18.5 mmHg, controls 121.2 ± 11.2 mmHg, CAD patients 113.9 ± 17.1 mmHg; p ≤ 0.0001*). There was also 1 vWD patient who had a diagnosed CAD in his anamnesis.

**Table 2.** Patient Characteristics.

	**vWD**	**n**	**Controls**	**n**	**CAD**	**n**	**p**
**Number of test persons (n)**	37		14		38		
**Gender**							0.000063*
Male [%]	57		43		95		
Female [%]	43		57		5		
**Age** [mean ± sd]	52.0 ± 9.3	37	49.1 ± 6.6	14	51.2 ± 4.4	38	0.434
**Body mass index** [kg/m^2^] [mean ± sd]	27.1 ± 7.1	31	24.7 ± 2.5	14			0.309
**Blood pressure** [mean ± sd]							
Systolic blood pressure [mmHg]	134.4 ± 18.5	30	121.2 ± 11.2	13	113.9 ± 17.1	38	< 0.0001*
Mean arterial pressure [mmHg]	99.5 ± 12.3	30	92.7 ± 6.0	13	84.5 ± 10.5	38	< 0.0001*
	**vWD [%]**		**Controls [%]**				
**Cardiovascular risk factors**							
Hypertension	38	37	0	14			0.019*
Type 2 diabetes mellitus	8	37	0	14			0.666
Smoking	19	30	29	14			1.000
**Infectious diseases**							
HIV	0	37	0	14			
Hepatitis	14	37	0	14			0.357
- Chronic hepatitis B	5	37	0	14			
- Diagnosed with hepatitis B, but no current disease	8	37	0	14			
- Chronic hepatitis C	0	37	0	14			
- Diagnosed with hepatitis C, but no current disease	8	37	0	14			
**Known CAD**	3	37	0	14	100	38	0.000*

CAD coronary artery disease, dl deciliter, HIV human immunodeficiency virus, mmHg torr, mg milligram, n number of test persons, p p-value, sd standard deviation, vWD von Willebrand disease, vs versus.

The statistical analysis of the biochemical markers of endothelial dysfunction are presented in Table 3. The sera of 3 vWD patients were inadvertently not frozen. Therefore, the MOED analysis could only be performed on 34 of 37 vWD patients.

**Table 3.** Results of the Markers of Endothelial Dysfunction.

	**sICAM-1** [pg/ml]	**P-Selectin** [pg/ml]	**Il-6** [pg/ml]	**TNF-α** [pg/ml]	**MCP-1** [pg/ml]
**vWD** [mean ± sd]	24988 ± 7584	7941 ± 3295	2.061 ± 2.179	0.273 ± 0.078	86.947 ± 67.351
n	34	34	34	34	34
**Controls** [mean ± sd]	21987 ± 4209	7307 ± 2163	1.141 ± 0.358	0.298 ± 0.095	80.950 ± 43.729
n	14	14	14	14	14
**p**	0.171	0.512	0.734	0.350	0.761

Il-6 interleukin-6, MCP-1 monocyte chemoattractant protein-1, ml milliliters, n number of test persons, p p-value, pg picogram, sd standard deviation, sICAM-1 soluble intercellular adhesion molecule-1, TNF-α tumor necrosis factor alpha, vWD von Willebrand disease.

The markers sICAM-1, P-Selectin, Il-6 and MCP-1 showed higher levels in patients with vWD but were not significant compared to the control group.

**Figure 1.**
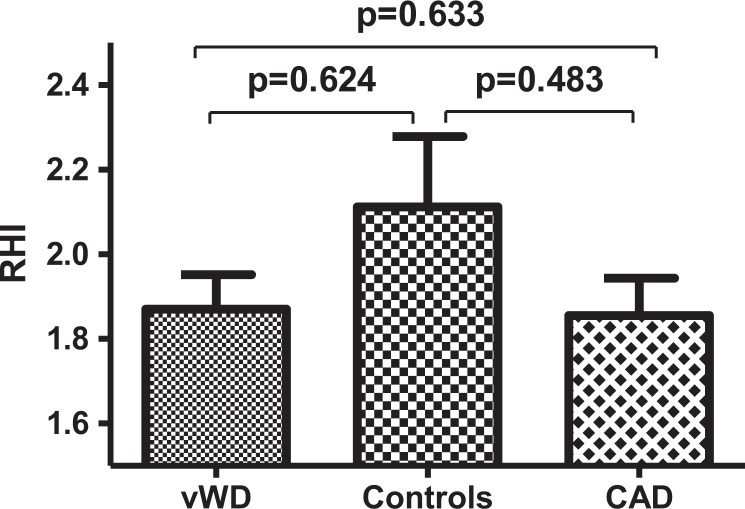
Reactive hyperemia index: vWD patients compared to controls and CAD patients (mean with standard error of the mean (SEM)).

The results of the RHI were not significantly different between the 3 groups (p = 0.367) (Table 4, Figure 1). The result of the RHI in patients with vWD (mean ± sd: 1.870 ± 0.446) was not significantly different to the control group (mean ± sd: 2.112 ± 0.601) (p = 0.624). The RHI was only measured in 30 vWD patients and 13 controls, as 7 vWD patients and 1 control person did not want to have another appointment for the RHI measurement. The 38 CAD-patients had as expected the lowest RHI with 1.855 ± 0.547 (mean ± sd; vWD versus CAD p = 0.633; controls versus CAD p = 0.483). In this study, the mean RHI of patients with vWD is located between the one of CAD-patients and controls.

**Table 4.** Results of the Reactive Hyperemia Index.

	vWD	Controls	CAD	p
**RHI** [mean ± sd]	1.870 ± 0.446	2.112 ± 0.601	1.855 ± 0.547	0.367
n	30	13	38	

CAD coronary artery disease, n number of test persons, RHI reactive hyperemia index, sd standard deviation, vWD von Willebrand disease.

In this study, the patients with vWD were divided again into subgroups of different severity levels. The 3 severity levels were severe (≤15% VWF activity), moderate (>15 to ≤30% VWF activity) and mild (>30 to ≤60% VWF activity). The classification was based on the lowest VWF activity measured in the hemophilia center and the severity specified in the medical letter. The mean VWF activity of patients with severe vWD (n = 14) was 8.443% ± 6.426, with moderate vWD (n = 5) 24.14% ± 4.530 and mild vWD (n = 18) 41.02% ± 7.018.

Table 5 shows the descriptive statistics of study results from the RHI and the 5 markers of endothelial dysfunction in patients with vWD after classification into the three severity levels. For RHI, the mean increased from the light vWD to the severe vWD group. As described in the introduction, the higher the RHI, the better the endothelial function. So, the tendency would suggest that the lower the VWF activity, the better the endothelial function. However, the differences between the groups were very small and not significant. Furthermore, in the multiple regression analysis there was no correlation between RHI and VWF activity (r = 0.07).

**Table 5.** Results of vWD Subgroup Analysis.

	RHI	sICAM-1	P-Selectin	Il-6	TNF- α	MCP-1
**Severe**						
n	10	13	13	13	13	13
mean ± sd	1.919 ± 0.576	24633 ± 10549	7159 ± 3543	2.351 ± 2.570	0.287 ± 0.051	70.238 ± 57.957
**Moderate**						
n	5	5	5	5	5	5
mean ± sd	1.904 ± 0.445	25337 ± 3785	9070 ± 2084	3.064 ± 3.188	0.316 ± 0.107	89.920 ± 69.860
**Light**						
n	15	16	16	16	16	16
mean ± sd	1.827 ± 0.371	25168 ± 5790	8223 ± 3412	1.511 ± 1.313	0.248 ± 0.082	99.594 ± 74.669

Il-6 interleukin-6, MCP-1 monocyte chemoattractant protein-1, n number of test persons, RHI reactive hyperemia index, sd standard deviation, sICAM-1 soluble intercellular adhesion molecule-1, TNF-α tumor necrosis factor alpha.

Subsequently, the RHI results in the three severity groups were compared with those in the control and CAD groups. Since the CAD group was not normally distributed, the Kruskal-Wallis test was used. In this global test p was 0.077. As a post hoc test, multiple Conover-Iman comparisons with p-Bonferroni-Holm correction were used. The results of the individual subgroup comparisons can be found in Table 6. The results showed that the RHI values of the subgroups did not differ significantly from those of the control group or the CAD group.

**Table 6.** Reactive Hyperemia Index: vWD Subgroups Compared to Control and CAD Group.

	p with Bonferroni-Holm correction
Control group versus:	
Severe vWD	1
Moderate vWD	1
Light vWD	1
CAD group versus:	
Severe vWD	0.595
Moderate vWD	0.835
Light vWD	0.684

CAD coronary artery disease, p p-value, vWD von-Willebrand disease.

Correlations between the results of the Endo-PAT and the MOED within the vWD group are shown in Table 7. No correlations were found between the RHI and the MOEDs.

**Table 7.** Correlations Within the Results of Patients With vWD.

	**RHI** p(r)
**sICAM-1**	0.433 (-0.15)
**P-Selectin**	0.051 (-0.37)
**Il-6**	0.297 (-0.2)
**TNF-α**	0.892 (-0.03)
**MCP-1**	0.355 (-0.18)

Il-6 interleukin-6, MCP-1 monocyte chemoattractant protein-1, p p-value, r Spearman’s Rank Correlation Coefficient, RHI reactive hyperemia index, sICAM-1 soluble intercellular adhesion molecule-1, TNF-α tumor necrosis factor alpha, vWD von Willebrand disease.

## Discussion

To the best of our knowledge, this is the only study where biochemical parameters combined with functional measurements in the form of RHI by using the Endo-PAT were measured to assess endothelial function in patients with vWD.

In this study, there were no significant differences in the comparison between control group, CAD-patients and vWD-patients what may be due to the fact that mainly patients with mild vWD have been included. Therefore, the vWD group was divided into subgroups determined by three severity levels. The Endo-PAT results and endothelial dysfunction markers were analyzed again and the RHI compared with the control and CAD group. Also, this analysis showed no significant results.

The RHI is even lower in vWD patients compared to the control group which would indicate a possible higher cardiovascular risk in vWD patients, but the differences are not great enough to draw conclusions.

According to a study from Bonetti et al. a RHI of <1.35 detects patients with coronary endothelial dysfunction with a sensitivity of 80% and a specificity of 85%.^16^ Matsuzawa et al. compared the RHI of women with obstructive CAD (median RHI: 1.57) and nonobstructive CAD (e.g. positive acetylcholin provocation test; median RHI: 1.58) with the RHI of women without ischemic heart disease (median RHI: 2.15). The study showed that at an index of 1.82, RHI results were 80% sensitive and 80% specific for identifying ischemic heart disease (obstructive and nonobstructive CAD).^17^

In the study carried out here no significant difference in RHI between CAD patients and controls was found. This is especially noticeable. However, the power of the performed Kruskal-Wallis-Test between the groups vWD patients, controls and CAD patients was only 22.82%. So, it must not mean that the validity of Endo-PAT has to be questioned. There would probably be a greater difference if the groups and the test strength were larger. No power analysis was performed before the start of the study to define the required group size as the study was designed as a pilot study.

The MOEDs sICAM-1, P-Selectin, Il-6 and MCP-1 were higher in vWD patients compared to healthy controls in this study which would also possibly indicate more endothelial dysfunction. However, the power of the performed tests between the groups vWD patients and controls was only 5%. A recently published study by Govorov et al. determined the levels of sICAM-1 and IL-6 as part of a study of the effects of the menstrual cycle on the clotting and immune system of female vWD patients.^18^ Also in this study, higher levels of sICAM-1 and IL-6 were measured in patients with vWD compared to healthy women.^18^

For the patients with vWD who were examined in the hemophilia center in the context of this study, the results overall showed no better endothelial function than in the control group. Thus, it is possible that the congenital bleeding tendency, in which especially the platelet aggregation is disturbed, does not have a protective effect against atherosclerosis.

In 2007, the results of a study on endothelial function in patients with vWD were published at the University of Padua, Italy. Endothelial function was measured by detection of flow-dependent dilatation (FAD) in the brachial artery. In this study, 24 patients with vWD type 2B and 24 control subjects were examined. The patients with vWD were on average 48 years and the controls 50 years old. After reactive hyperemia, the diameter of the brachial artery by FAD increased by 15.2 ± 3.1% in the vWD patients and by 14.1 ± 2.9% in the control group. There was no significant difference between the 2 groups. The intima-media thickness of the carotid, brachial and femoral artery and abdominal aorta was also measured by B-mode ultrasound. The analysis of the measurements showed no significant difference between the 2 groups.^19^

In a study from the Netherlands by Zwiers et al. from 2013 with 26 vWD patients and 52 controls it was found that atherosclerosis was similar in patients with and without von Willebrand syndrome.^20^

Looking at several studies on atherosclerosis in patients with vWD, the literature is inconclusive about the protective effect of VWD on atherosclerosis. A detailed analysis of these studies is described in the review by Van Galen.^10^

In an old report from 1983 cases of coronary artery disease in patients with vWD are described.^21^ Some more cases are summarized by Girolami et al.^22^

In vWD patients, the studies presented here and the results of this work suggest that vWD patients exhibit no significantly reduced levels of endothelial dysfunction and atherosclerosis compared to the general population. Thus, their risk for coronary artery disease does not seem to be lower.

Though, a lower mortality rate cannot be ruled out despite no reduced cardiovascular risk factors, endothelial dysfunction and degree of atherosclerosis because thrombus formation after plaque rupture could be reduced.^9^

The low number of reported coronary artery disease in vWD patients makes the impression of a low incidence. Studies examining the morbidity and mortality rate for CAD in the current vWD population, who have a similar life expectancy to the general population, would be necessary in this context. One relatively new retrospective analysis of the discharge data of adults from the Nationwide Inpatient Sample between the years 2009 and 2011 in the US has been done by Seaman et al.^23^ The results showed a relatively lower prevalence of CAD in vWD patients compared to non-VWD patients (15.0% versus 26.0%).^23^

However, vWD patients are not entirely protected from atherosclerosis. Therefore, a focus should still be laid on this age-related comorbidity in the aging vWD population. The literature shows that there are vWD patients who have developed coronary artery disease. Based on this finding, it could be recommended that vWD patients should receive the same lifestyle advice for the prevention of coronary artery disease as well as pharmacological therapy to treat cardiovascular risk factors. Moreover, it seems useful to implement treatment strategies for CAD that are consistent with the increased risk of bleeding in vWD patients.

Limitations of this study were the small number of observations for controls. Therefore the vWD and control group were not matched in numbers (37 vWD vs 14 controls, of which some drop out in the Endo-PAT measurements). The mean VWF activity level in the subgroup of type 1 patients was 36.2 ± 11.8%. The question arises whether a stricter definition of vWD type 1 would have changed the results of the study. Data on VWF levels for controls and CAD patients has not been collected. Thus, in these patients a vWD was not excluded. Given that low VWF has been reported in a great number of the general population, and that high VWF levels are associated with high risk of cardiovascular disease, it would have been essential to know what the levels are in the control and CAD groups of the study. Also gender imbalance probably reduced the informative value of the study. The CAD group was almost entirely male whereas the others were about 50:50 male and female. The mean age of vWD patients was 52 years. Endothelial dysfunction precedes clinically apparent CAD, but a protective effect of their bleeding disorder might better be analyzed in an older study population. Furthermore, no information of drug treatment was collected. The CAD group will clearly be receiving drugs for cardiovascular disease, which may have an impact on the results. One patient with vWD also reported to have CAD and probably received drugs for cardiovascular disease. All measurements were performed accurately, however, measuring errors might still be possible. Subgroup analysis of vWD patients with and without hepatitis were not performed, because only 5 patients with vWD had different forms of hepatitis. Therefore, possible influences by hepatitis B and C cannot be ruled out.

In conclusion, we hypothesize that vWD patients exhibit no reduced levels of endothelial dysfunction and therefore have a similar risk for coronary artery disease as the general population. However, the mortality rate for CAD could still be positively affected by their congenital bleeding disorder because thrombus formation after plaque rupture may be reduced.
